# Differences in barriers for controlled learning about safety between biotechnology and chemistry

**DOI:** 10.1038/s41467-022-31870-8

**Published:** 2022-07-14

**Authors:** Britte Bouchaut, Frank Hollmann, Lotte Asveld

**Affiliations:** 1 Section of Biotechnology and Society, Department of Biotechnology, Delft University of Technology, Van der Maasweg 9, 2629HZ Delft, The Netherlands; 2 Section of Biocatalysis, Department of Biotechnology, Delft University of Technology, Van der Maasweg 9, 2629HZ Delft, The Netherlands

**Keywords:** Biotechnology, Decision making, Green chemistry

## Abstract

In contrast to chemical industry, biotechnology is still not competitive for the production of chemicals, materials, and biofuels. Here, the authors discuss the underlying reasons and propose to address the problem through regulatory changes and risk management.

## Teaser

The increasing societal demand for safer, biobased products, and processes creates opportunities for industrial biotechnology and chemistry. To succeed, controlled learning about new emerging risks is crucial but both fields endure difficulty in doing so by their respective regulation and risk management culture.

The planetary boundary for production and release of new chemicals and plastics^1^, rising CO_2_ levels, depletion of fossil-based raw materials, and geopolitical dependencies present an urgent call for an industrial transition toward a biobased economy. Industrial biotechnology (and the associated field of green chemistry) aim to find more sustainable alternatives to conventional chemical manufacturing routes. Particularly the development of CO_2_-negative approaches (e.g., CO_2_ conversion into chemicals and fuels^2^) and biobased alternatives to fossil resources-derived chemicals, polymers, and plastics^3,4^ show great potential to fight today’s problems and for countries or regions to become less dependent on others. However, biotechnology is struggling to compete with conventional chemical methodologies^5,6^. This can be explained by the history, size^7,8^, and influence (e.g., having a strong lobby in terms of policy measures^9,10^) of the chemical industry, and the simple fact that these industries are already established and matured compared to the biobased industries. However, it also appears that the respective risk management cultures in each industry differ greatly, which hinders the development of biotechnology and the biobased industry in becoming technically and economically feasible.

The risk management culture in biotechnology emphasizes uncertain risks and is subject to a strong precautionary regime, particularly in Europe, leaving little room for development when uncertain risks are involved. In contrast, for chemistry, the focus is on known risks which has resulted in a culture of passive learning (i.e., through accidents) and many examples of regrettable substitution^11,12^. The two risk management regimes seem to be at odds with each other even though both types of risk emerge in each field. If we want to tackle the global challenges of today, we need to develop new, safer products and processes that may require new types of chemistry in which biotechnology could play a pivotal part. This requires a middle way between the risk management regimes of chemistry and biotechnology: one that stimulates awareness of uncertain risks and also creates room to gain new knowledge of these risks. Therefore, we need to put designated procedures and institutions in place solely for the aim of learning about uncertain risks, i.e., active, or controlled learning, so new products and processes can be developed safely. Safe by Design (SbD) approaches could provide a framework to achieve such controlled learning, as has already been demonstrated in biotechnology^13^ and nanotechnology^14^. SbD is an emerging approach that entails adaptive and iterative risk management by providing strategies to make researchers and research institutions question the initial usage of (possibly) hazardous compounds and/or encourages to (completely) rethink a technology’s design process already during the very early stages of development (e.g., during R&D) for the sake of safety^13,15^. Thereby, the approach focuses on learning what possible risks might emerge and encourages alternative design choices to circumvent the earlier identified emerging risk. This is no guarantee that safety is ensured, but it does place more emphasis on designing for safety. Therefore, we propose it to also be implemented in the conventional chemical industry—as we will elaborate in this article. In addition, although we focus on safety, other notions such as circularity can also be included when considering design choices (i.e., Safe and Circular by Design^16^).

## Known and uncertain risks

While societal concerns have had consequences for regulation and managing risks in both industries, each field’s respective regime places emphasis on a different type of risk. This seems to be at odds with each other as both uncertain and known risks emerge in either field. With uncertain risks, we refer to risks that are not completely known, for instance, it might not be known what the order of magnitude is of a possible detrimental effect, or it might not be known what the possible detrimental effects are to begin with^17^.

The field of industrial biotechnology is associated with known and particularly with uncertain risks. In terms of known risks and in response to public concerns, measures such as containment have been taken to lower or mitigate these risks. In terms of uncertain risks, applications such as CRISPR are still under development and can give rise to possible issues such as mutations or off-target effects^18^ that are difficult and complex to identify and anticipate. Other forms of engineering organisms and subsequently applying them in industrial processes also give rise to uncertain risks. Strategies for reducing and anticipating risks for these types of applications are well developed, e.g., by auxotrophy^19^ or building in a conditional dominant lethal gene^20^. Even though these strategies may not be perfect^21,22^, they do show that the field is actively dealing with uncertain risks.

In contrast, the chemical industry relies strongly on existing knowledge of risks. Chemical engineering’s history as a scientific discipline goes back to the early 18th century and since then, many incidents have occurred. Therefore, there is vast knowledge and experience of the tragic consequences of these incidents (i.e., through passive learning) such as global pollution by micro-plastics^23^ or the widespread occurrence of PFAS^24,25^. While part of the industry has devoted itself to designing safer products and processes by utilization of the green chemistry principles^26^ or SbD strategies^27^, still there are many reported cases of regrettable substitution^27,28^—replacing a hazardous chemical with an alternative that is suitable in technical and economic terms, but just as harmful or potentially worse as the replaced chemical. Here we particularly emphasize regrettable substitutions by negligence (e.g., PFAS^29^), which often appeared to have been induced by the conventional chemical industry. Despite calls for a more ethical, greener chemistry^9,30,31^, the latter illustrates that this part of the industry has not been able, or unwilling, to deal with known or uncertain risks effectively.

## Risk management in biotechnology and chemistry at odds

Each discipline’s respective risk management approach provides little room or incentive to learn what uncertain risks entail. Europe’s highly precautionary regime in biotechnology results in a culture of compliance^32^ meaning that when no conclusive evidence can be provided that an emerging uncertain risk would be acceptably safe, innovations might be put on hold until safety can be guaranteed. In chemistry, managing risks is based on conclusive evidence that a new product or application is not safe, creating little incentive for the conventional chemical industry to actively research uncertain risks (or provide data concerning known risks) as this can lead to their new technology becoming prohibited or market entry postponed.

For clarity, this next section focuses mostly on differences in regulation between either field in Europe, and differences between Europe and the US. However, the problems we face today illustrate that risk management in biotechnology and chemistry is of importance on a global scale. The sections below can also provide insights for regulation in other parts of the world.

Regulation concerning chemicals in both Europe and the United States actively promotes and calls for the progressive substitution of the most dangerous chemicals when suitable alternatives have been identified. Although this, in theory, seems solid regulation to increase safety, several problems have been encountered. While a precautionary approach has been embedded in US regulation concerning chemicals (i.e., TSCA), the approach’s operationalization fails to lead to higher safety. Mostly as current legislation in the US calls for conclusive evidence that a new product or process cannot be considered safe for it to be banned or strictly regulated. This results in little incentive for the conventional industry to test on safety of the vast majority of chemicals. On the other hand, a small subset of chemicals is subjected to a highly precautionary culture but has resulted in “an inequitable barrier to entry for newer, safer chemicals”^33^. It discourages industry to develop new and possibly safer chemicals, chemical products, and processes as information about new risks could be used against them in a later stage^33^. This also relates to other regulatory problems such as the conventional industry filing incomplete dossiers, necessary information not being available due to confidentiality issues^34,35^, or issues concerning regrettable substitution (by negligence). Particularly the latter illustrates the passive learning, or learning-by-doing aspect, of which the so-called “forever chemicals” or “Generation-X chemicals” are the most illustrative^25,28,36^.

For European regulation (i.e., REACH)^28^, has already called for regulatory changes to tackle regrettable substitution and point out several reasons why this is still an ongoing problem. The main reasons they put forward are (1) absence of information regarding hazard properties of the substitute substance, (2) inconsistencies in the implementation of the European Chemicals Regulations, and (3) lack of interest of some part of the industry to manage stringent classifications. In addition, incomplete dossiers and necessary information not being available due to confidentiality issues^34,35^ lead to the scenario of “no data, no problem”^28^ where the industry seems to be working toward innovating for circumventing existing environmental norms and legislation, instead of working on truly safer alternatives. Lastly, regulation calling for the progressive substitution of dangerous chemicals when suitable alternatives have been identified gives rise to another problem. To find safer alternatives to hazardous compounds, industry has to engage in active research. However, incentives appear to be lacking. As already referred to with the issue of regrettable substitution, economic and technical feasibilities are given great value instead.

In contrast with chemicals, biotechnology is regulated more strictly. In Europe particularly, biotechnology is regulated based on precaution which gives rise to a completely different way of handling risks. First of all, in terms of allocated responsibility, initial stakeholders (researchers/engineers, companies) are responsible for providing conclusive evidence that their experiments and innovations only involve acceptable risks, and thus can be deemed safe. But, in terms of managing uncertain risks, this responsibility lies with risk managers and assessors and ultimately the government^32^. This results in a culture of compliance: if there are uncertain risks involved, one has to redesign the technology or process to comply with the set norms^37^. While for chemicals, the responsibility for managing uncertain risks is allocated to the industry which does not incentivize them to provide data, nor comply with the norms when uncertainties are involved.

Both US and EU regulations regarding biotechnology value safety and therefore regulation is strict. But US regulation does differ in having a product-based assessment instead of process-based in Europe. Thereby, in the US, the innovative character of biotechnology is more emphasized. Since 2019, the USDA has implemented exemptions for GE crops that could have also been produced through conventional breeding techniques. This change now allows crop developers to self-determine whether such an exemption applies to their product, but does not influence the outcome of the USDA’s review process for crops created by GE techniques (Regulatory Status Review). The motivation for this regulatory change was to stimulate innovation and make governmental oversight more effective and efficient^38,39^. Also, this change has contributed to leveling the playing fields between conventional breeding and crop improvement using biotechnology—crops with the same outcome should not be regulated differently (product-based assessment). However, it appears that this revised regulation for GE crops has also led to businesses avoiding disclosure of e.g., methods and genes due to confidentiality issues^40^—which might give rise to new “no data, no problem” issues we already know from chemistry.

Although US regulation allows more room for innovation in biotechnology compared to the EU, this is no guarantee that as a result the conventional chemical industry will adopt products and processes derived from biotechnology. As mentioned, the conventional industry shows a lack of incentive and seems to be mostly profit driven. While green chemistry can also be profitable, this would still require substantial investments, differently set-up research, development, and implementation. Therefore, as long as changes are not enforced, it is plausible that the conventional industry will stick to what they have been doing for many years.

## Controlled learning about uncertain risks

It has become clear that the risk management cultures in biotechnology and chemistry either do not provide much room or incentivize learning what uncertain risks entail. For the sake of safe and responsible development of new products and processes, regimes where a culture of passive learning prevails (i.e., the conventional chemical industry) need to become one of active, controlled learning^41^. And regimes in which learning is currently stifled (mostly pertaining to European biotech regulation), regulation should change to allow room for such learning about uncertain risks. To enable active, controlled learning in all industries, SbD could provide a suitable framework.

SbD is an adaptive and iterative risk management approach that focuses on learning what possible risks might emerge and encourages alternative design choices to circumvent the earlier identified emerging risk. Depending on how much room regulation allows for uncertain risks, these strategies are based on mitigating or lowering known risks or can be applied to gradually learn in a step-by-step way what uncertain risks entail. As mentioned before, controlled learning about uncertain risks through SbD needs designated procedures and institutions to be put in place specifically for the aim of learning about uncertain risks^41^. Not only would this require organizations and research to be set up differently but also a culture change. The latter will be very hard to accomplish without incentives that provide a shift from economic motives to safety. In that sense, this would require (1) changes in the chemical industry i.e., higher attention to safety by enforcement, holding companies accountable for damage and stimulating transparency, (2) changes in education and academic research, and (3) governmental measures in terms of policy adaptations.

First of all, to incentivize or enforce adoption of SbD-thinking in industry, developers should be made accountable for negative externalities resulting from their products or processes. Also, regulation could provide additional funding or research grants to companies to research safer alternatives. Partnerships could be stimulated to innovate and share information about new products with the industry hopefully sparking a broader adoption. Thereby, it could become more appealing for e.g., the conventional chemical industry to adopt SbD and actively work on the creation of safer products and processes.

Secondly, the “learning about uncertain risks” should be reflected in how research is being valued in awarding research grants. Currently, the supporting academic system (e.g., funding organizations, research institutions, and universities) does not seem to be very supportive of risk research as mostly technically innovative research is awarded. That way, we are missing out on important knowledge and data concerning potential risks in biotechnology and chemistry, and of which risk governance could benefit as well. In that sense, academic scholars should also embrace this different way of thinking to a greater extent and become more focused on risk research. And vice-versa, publishing agents (peer-reviewed academic journals) should value risk research the same as technical research. Also, we can work toward a culture wherein safety is embraced to a larger extent by targeting the “engineers of tomorrow” and embedding the “designing for safety” way of thinking already in education. To do so, knowledge institutions engaged in education should also devote part of their curriculum to the SbD-way of thinking—examples can be coming from iGEM (International Genetically Engineered Machines; https://igem.org/), a yearly student competition in synthetic biology that highly regards safety and SbD. Only through education, we can reach the future engineers and embed this way of thinking in future company—and industry cultures and risk management.

Still, bringing drastic change culture-wise can often not be achieved from a solely bottom-up initiation. Therefore, policy should no longer place the responsibility of managing uncertain risks with industry as the current allocation appears to be too tempting for misuse by part of the industry. Therefore, responsibility needs to be redistributed leaning toward a regime of compliance. That would mean that industry would be responsible for providing conclusive evidence that a new product or process is safe, thereby hopefully sparking an incentive to conduct more risk research. However, for that, total transparency would be needed—eliminating the current “no data–no problem” issue. Therefore, regulation needs to put requirements for transparency into place, not only concerning known risks but also uncertain risks. Also, society must have independent testing so that the provided information can be relied upon, and companies should be required to report on their use of chemicals and their efforts to reduce hazards (by SbD), and pay support systems that recover post-use products. Not only for the sake of safety itself but also for people and animals having the right to a healthy and safe living environment.
